# Deep Learning Neural Modelling as a Precise Method in the Assessment of the Chronological Age of Children and Adolescents Using Tooth and Bone Parameters

**DOI:** 10.3390/s22020637

**Published:** 2022-01-14

**Authors:** Maciej Zaborowicz, Katarzyna Zaborowicz, Barbara Biedziak, Tomasz Garbowski

**Affiliations:** 1 Department of Biosystems Engineering, Poznan University of Life Sciences, Wojska Polskiego 50, 60-627 Poznan, Poland; tomasz.garbowski@up.poznan.pl; 2 Department of Orthodontics and Craniofacial Anomalies, Poznan University of Medical Sciences, Collegium Maius, Fredry 10, 61-701 Poznan, Poland; biedziak@ump.edu.pl

**Keywords:** chronological age, dental age, age assessment, digital pantomography, digital image analysis, artificial intelligence, deep neural network

## Abstract

Dental age is one of the most reliable methods for determining a patient’s age. The timing of teething, the period of tooth replacement, or the degree of tooth attrition is an important diagnostic factor in the assessment of an individual’s developmental age. It is used in orthodontics, pediatric dentistry, endocrinology, forensic medicine, and pathomorphology, but also in scenarios regarding international adoptions and illegal immigrants. The methods used to date are time-consuming and not very precise. For this reason, artificial intelligence methods are increasingly used to estimate the age of a patient. The present work is a continuation of the work of Zaborowicz et al. In the presented research, a set of 21 original indicators was used to create deep neural network models. The aim of this study was to verify the ability to generate a more accurate deep neural network model compared to models produced previously. The quality parameters of the produced models were as follows. The MAE error of the produced models, depending on the learning set used, was between 2.34 and 4.61 months, while the RMSE error was between 5.58 and 7.49 months. The correlation coefficient R^2^ ranged from 0.92 to 0.96.

## 1. Introduction

Dental age is one of the most reliable methods for determining the maturity of an organism [1]. It is extremely useful in areas such as orthodontics, pediatric dentistry, endocrinology, anthropology, or forensic medicine [2,3,4,5,6,7,8,9]. It allows us to determine whether the body is developing properly and when a pubertal growth spurt occurs. Moreover, the dental age assessment can be used to determine the age of individuals without identification documents or those suspected of having falsified documents, with memory loss, illegal immigrants, or international adoptions [10,11].

Age determination using pantomographic radiographs is an easy, widely available, and low-cost method. In children, the developmental stages of tooth buds, mineralization of crowns and roots, and the eruption stages of teeth can be assessed [12,13,14]. In the elderly, changes in the dentition are not very noticeable, thus age assessment is much more difficult. However, it is possible to take advantage of the fact that, with age, odontoblasts deposit more and more secondary dentin, causing a reduction in pulp chamber volume. Methods that analyze the alveolar bone level have also been described [15,16,17].

The commonly used methods to determine dental age, such as Demirjian’s method, Schour and Massler’s method, Ubelaker’s method, Moorres’, Fanning and Hunt’s method, Noll’s method, or Gustafson and Koch’s method, are methods developed in the previous century [13,18,19,20,21,22]. The phenomenon of acceleration, or growth spurt, occurring in the population makes these methods inaccurate. Therefore, there are noticeable discrepancies between the chronological age and the age determined from the developed tables, atlases, and charts [23,24,25,26].

Determining the dental age based on tables and charts is also time-consuming and subjective. The doctor themselves must compare the degree of development of the buds of most teeth on the patient’s pantomographic image with the images presented in the studies.

Taking into account the limitations of the methods used to date and the individual variability of the human body, the search began for objective methods which do not require the involvement of a physician in the assessment of the patient’s age and which can cope with non-linear biological issues. Innovations in the field of computer science, including methods of artificial intelligence, are increasingly used in medicine. They support diagnosis and improve treatment efficiency [27,28,29,30,31,32,33,34,35,36,37,38,39,40,41,42,43,44,45].

In scientific databases such as Web of Science and Scopus, one can find many papers confirming the effectiveness and efficiency of artificial neural networks in dentistry, including the assessment of dental age.

The application of artificial neural networks in information and image processing in dentistry was presented by Kim et al. in 2021 [46]. They investigated the estimation of age groups by applying a conventional neural network (CNN) using X-ray images of first molars on pantomographic images. The data set consisted of images of maxillary and mandibular first molars on the right and left sides. In total, 1586 pantomographic images were used in the study. The conventional neural network produced was shown to focus on anatomical parameters such as the dental pulp chamber, alveolar bone level, and interdental space. The efficiency of the networks generated in this way was very high, ranging between 87.04 and 88.33%. It was also shown that there were slight differences depending on the location of the first molar.

The team of Farhadian et al. [47] presented another example of the use of artificial intelligence in the assessment of dental age. The study used 300 scans taken with cone beam computed tomography (CBCT) of individuals between 14 and 60 years. Researchers assessed the ratio between the dental pulp and the tooth. Additionally, a neural network model was compared with a linear regression model. The results presented show that the neural network model has a lower root mean square error (RMSE) of 4.40 years and mean absolute error (MAE) of 4.12 years, compared to the linear regression model, which had an RMSE of 10.26 years and an MAE of 8.17 years.

In contrast, the 2021 paper by Banjšak et al. [48] used deep convolutional neural networks to estimate the age group. The learning set consisted of 4035 pantomographic images. The developed neural network was used to estimate the age of 89 archaeological skull remains. The accuracy of the developed network is 73%.

Deep convolutional neural networks were also presented in the works of Milošević et al. [49] and Kahaki et al. [50]. They evaluated the accuracy of dental age estimation from X-rays. Milošević’s team created a learning set consisting of 4035 pantomographic radiographs and 76,416 dental radiographs of individuals aged 19–90 years. The median error was 2.95 years for panoramic images and 4.68 years for single tooth images. Kahaki’s team, on the other hand, evaluated the effectiveness of estimating a patient’s age using artificial intelligence using 456 pantomographic images of children between the ages of 1 and 17. They created 12 neural networks representing the age groups: 1–4, 5–7, 8–10, 11–13, 14–17, and 1–17 by male and female gender. The networks for the age group 14–17 for each gender have the highest test quality of over 90%. For the other age groups, the test quality was more than 80%.

One of the most recent papers on metric age assessment of children and adolescents on pantomographic radiographs is by Zabrowicz et al. [51]. They developed a set of 21 tooth and bone indicators and investigated whether it is possible to create a neural model to support the assessment of metric age. In this study, three models were generated: one for men and women, a separate one for women, and a separate one for men. The created artificial neural network model containing cases of men and women allows us to determine the metric age with a quality for the test set of 0.997394 and an error for the test set of 0.036526. On the contrary, the model containing cases of women only had a quality for the test set of 0.963090 and an error for the test set of 0.033634, while the test quality of the model determining the metric age of men was 0.999342 and the error for the test set was 0.039840.

Artificial neural network is an information processing system whose structure and operating principle resemble the information processing system in a human neuron. It is on biological inspiration that artificial neuron schemes and structure are based.

Currently, neural modeling is a method widely used by scientists and in industry. Neural networks are a computer tool that can solve complex problems without prior mathematical formalization.

Neural modelling is very popular method in the biological and medical community [52]. It can be used in many diagnostic aspects [53,54,55,56,57,58,59,60,61,62,63]. Increasingly, deep learning methods are being used to solve scientific problems. One simulator of deep neural networks is the H_2_O program [64,65,66]. The H_2_O software can be obtained for free from the H2O.ai website and used in accordance with the license. The project itself is Open Source. The application can be used via a web browser, e.g., on a local computer where H_2_O simulator is running. In this study, H_2_O simulator and Deep Learning method were used to generate new neural networks determining the metric age of children from 4 to 15 years old. The aim of this study is to check the possibility of creating accurate (as low as possible MAE and RMSE error, high R^2^ coefficient) models, which would allow to quickly and effectively determine the metric age of the examined patients on the basis of the provided data.

The present work is a continuation of the work of Zaborowicz et al. In the presented research, a set of 21 original indicators was used to create deep neural network models. The aim of this study was to verify the ability to generate a more accurate deep neural network model compared to models produced previously.

Ethical Statements: The Bioethics Committee of the Medical University of Poznań considered that the research carried out does not have the characteristics of a medical experiment and therefore agreed to carry out the relevant work.

## 2. Materials and Methods

### 2.1. Research Material and Methodology

The source of the analyzed data was the database of patients (children and adolescents aged from 48 to 144 months) of the University Centre of Dentistry and Specialist Medicine in Poznań, Poland. The research material consisted of 619 digital pantomographic images (296 photos of girls and 323 photos of boys). All analyzed cases were verified, and photographs which presented abnormalities or developmental disorders were excluded. Additionally, it should be added that experiments were not performed on children. The Bioethics Committee of the Medical University of Poznań considered that the research carried out does not have the characteristics of a medical experiment and therefore agreed to carry out the relevant work.

The following research methodology was used in this study:

1. Acquisition of research material-pantomographic images of children and adolescents aged 4 to 15 (from 48 to 144 months);
2. Verification and exclusion of abnormal cases and preparation of a database of selected digital pantomographic images;
3. Determination of patients’ age at the moment of picture taking, expressed in months;
4. Determination of a set of tooth and bone parameters;
5. Collection of tooth and bone parameters using ImageJ software;
6. Definition of a set of indicators, i.e., values of proportions of measured tooth and bone parameters;
7. Preparation of a learning set for neural modelling;
8. Neural modelling in H_2_O.ai;
9. Verification of the produced models;
10. Comparison of models with models produced in STATISTICA 7.1 simulator.

### 2.2. Methodology for Obtaining Empirical Data—New Tooth and Bone Indicators

**Figure 1:**
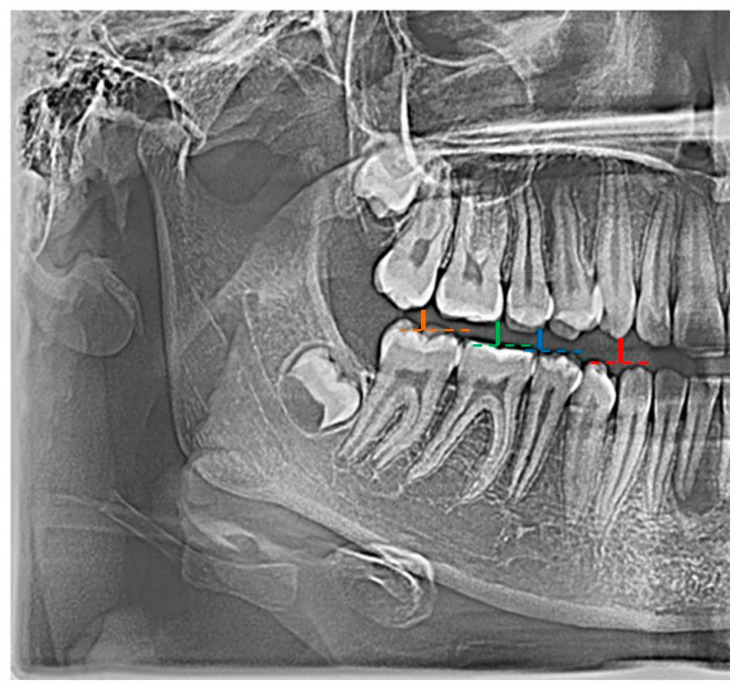
Example graphical representation of indicators: X01 (color: red |C13C43|; blue |C15C45|), X02 (color: red |C13C43|; green |C16C46|), and X03 (color: red |C13C43|; orange |C17C47|).

In the conducted research, an original and authored set of 21 indicators was used, i.e., distinctive tooth and bone parameters, which were developed in the form of mathematical proportions X01–X21 by Zaborowicz [51] (Figure 1).

### 2.3. Research Methods

The pantomographic photos used in the research were taken with the Duerr Dental-VistaPano S Ceph camera which was equipped with an X-ray head with 0.5 mm focus and a digital sensor, Cls-CMOS matrix in DICOM 3.0 format supported by DBSWIN [67]. The measurements of tooth and bone parameters were performed in Open Source software ImageJ 1.52a [68]. Additionally, MS Excel 2007 spreadsheet was used to aggregate and structure the data obtained in the process of image processing and analysis, which also enables saving the data in *.csv format [69].

The process of generating a neural model was carried out using H_2_O.ai. software (version 3.24.0.5) with Deep Learning methods, which allows us to create, validate, and predict artificial neural network models. In this software, it is also possible to perform a sensitivity analysis of variables of the developed models [64,65,66]. Deep learning is a class of machine learning methods for hierarchical (deep) models with nonlinear layers [70]. The idea of deep learning is to pretrain the network, and in the next step to train the network in a supervised manner—this method can combine supervised and unsupervised learning. In order to carry out the learning process properly, a large dataset is usually required; however, this is not necessary due to the deep neural network’s performance, which has the ability to redundancy. In brief, it can be said that the network “breaks” data into smaller parts and, on the basis of these smallest elements, aims to generalize the processed information.

## 3. Results

Three deep neural network models were generated during the study: one for the learning set of women and men, and one each for the learning set of women and the learning set of men. During the modeling process, all 21 new indicators and the gender indicator were used [51]. After each model was generated, predictions were made for each entire learning set. The learning set of women and men contained 619 samples; the learning set of women contained 296 samples; and the learning set of men contained 323 samples. A sensitivity analysis of the variables was also conducted for each of the models that were generated.

The models were characterized by the following parameters: MSE (Mean Squared Error) Equation (1); RMSE (Root Mean Squared Error) Equation (2); R^2^ (R Squared); MAE (Mean Absolute Error) Equation (3); MAPE (Mean Absolute Percentage Error) Equation (4); and RMSPE (Root Mean Squared Percentage Error) Equation (5).

(1) $$\mathrm{MSE}=\frac{1}{N}\overset{N}{\underset{i=1}{\sum }}{\left(t_{i}-y_{i}\right)}^{2}$$

(2) $$\mathrm{RMSE}=\sqrt{\frac{1}{N}\overset{N}{\underset{i=1}{\sum }}{\left(t_{i}-y_{i}\right)}^{2}}$$

(3) $$\mathrm{MAE}=\frac{1}{N}\overset{N}{\underset{i=1}{\sum }}\left|t_{i}-y_{i}\right|,$$

(4) $$\mathrm{MAPE}=100\frac{1}{N}\overset{N}{\underset{i=1}{\sum }}\left(1-\frac{y_{i}}{t_{i}}\right)$$

(5) $$\mathrm{RMSPE}=100\sqrt{\frac{1}{N}\overset{N}{\underset{i=1}{\sum }}{\left(\frac{t_{i}-y_{i}}{t_{i}}\right)}^{2}}$$

### 3.1. Model to Determine Metric Age for Men and Women

The parameters representing the quality of the generated models for the learning set of male and female are presented in Table 1.

This means that the mean MAE prediction error was 4.61 months. Additionally, MAPE and RMSPE parameters were calculated, respectively, as 4.10% and 6.36%.

**Figure 2:**
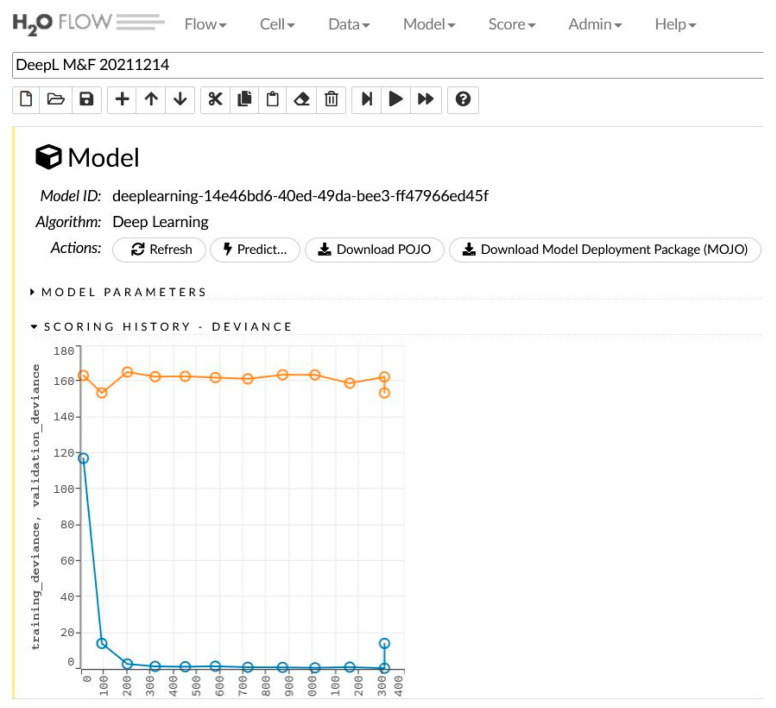
The generated model for women and men and the learning process.

**Figure 3:**
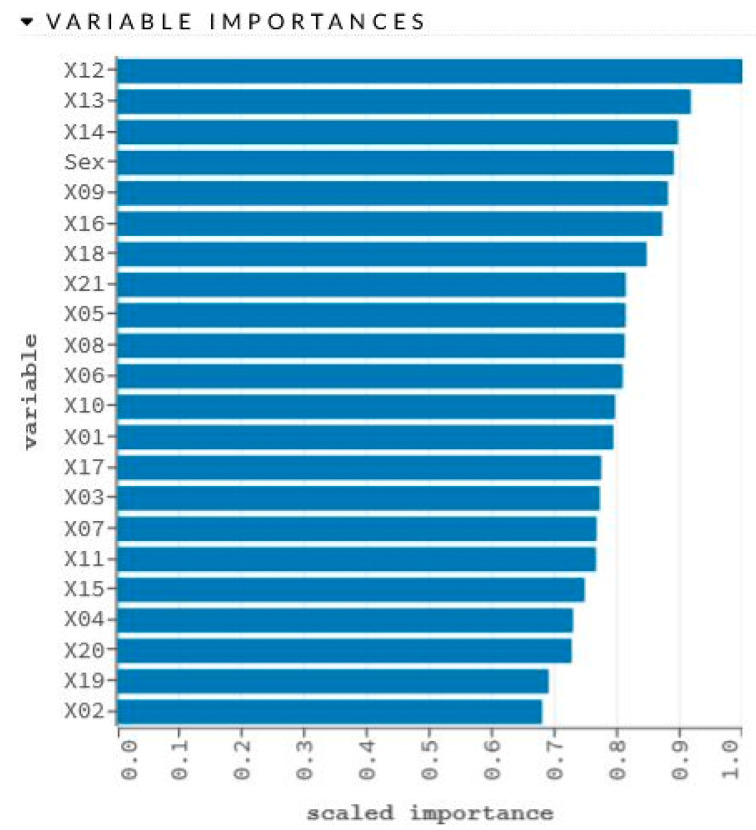
Graphical representation of sensitivity analysis of variables.

The network learning process is in Figure 2. The sensitivity analysis is shown in Table 2 and Figure 3.

### 3.2. Model to Determine Metric Age for Women

The parameters representing the quality of the generated models for the learning set of male and female are presented in Table 3.

This means that the mean MAE prediction error was 3.85 months. Additionally, MAPE and RMSPE parameters were calculated, were, respectively: 3.48% and 6.86%.

**Figure 4:**
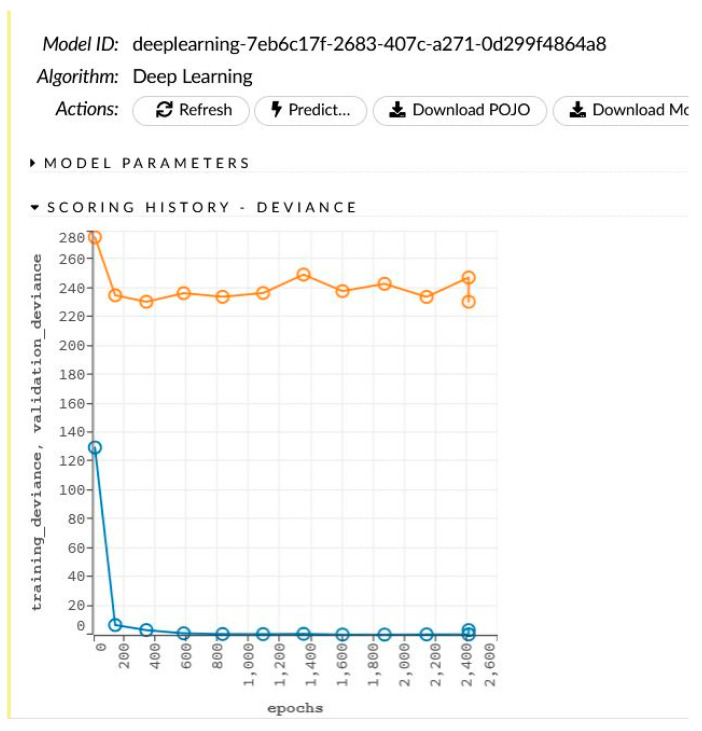
The generated model for women and the learning process.

**Figure 5:**
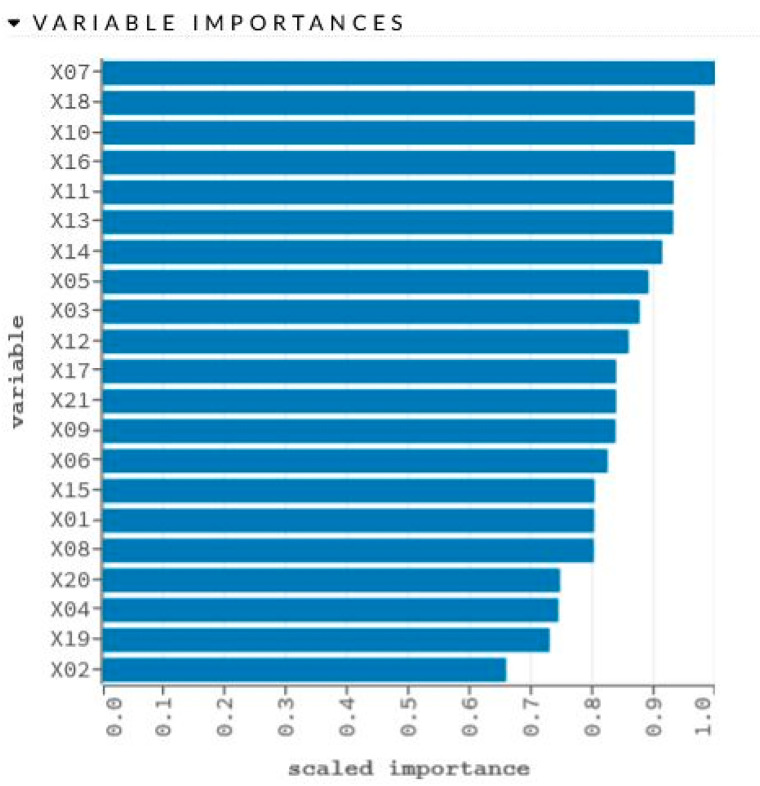
Graphical representation of sensitivity analysis of variables.

The network learning process is in Figure 4. The sensitivity analysis is shown in Table 4 and Figure 5.

### 3.3. Model to Determine Metric Age for Men

The parameters representing the quality of the generated models for the learning set of male and female are presented in Table 5.

This means that the mean MAE prediction error was 2.34 months. Additionally, MAPE and RMSPE parameters were calculated, were, respectively: 2.04% and 4.83%.

**Figure 6:**
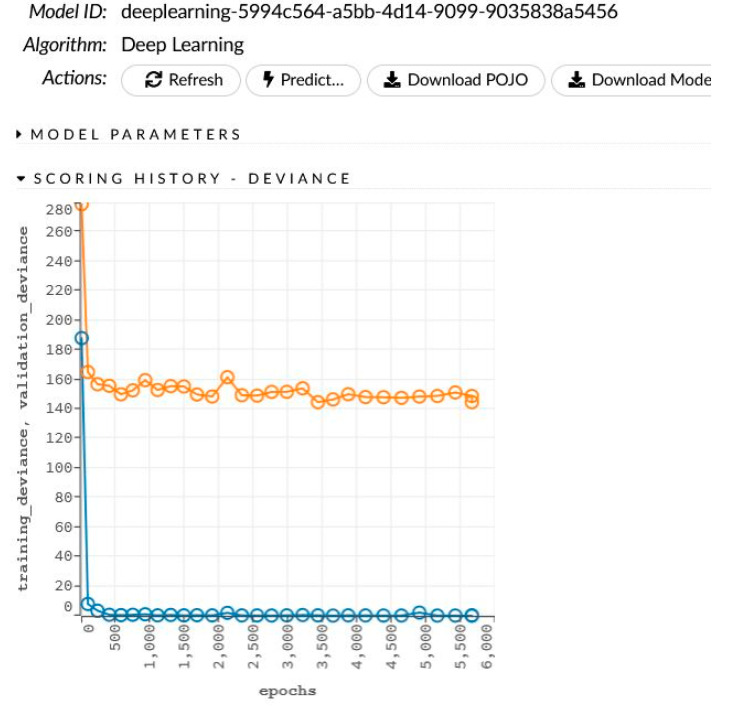
The generated model for women and the learning process.

**Figure 7:**
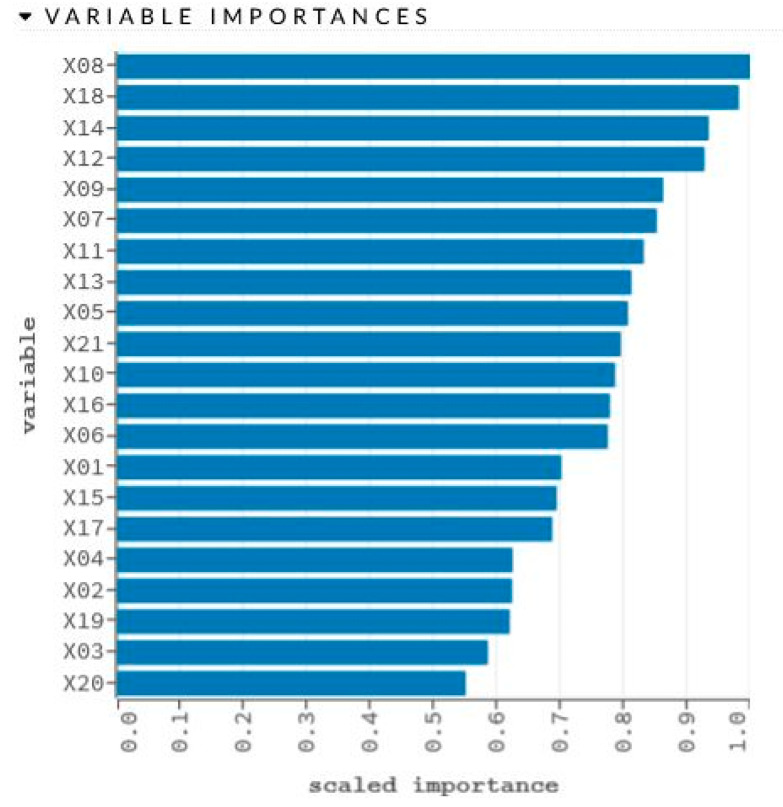
Graphical representation of sensitivity analysis of variables.

The network learning process is in Figure 6. The sensitivity analysis is shown in Table 6 and Figure 7.

## 4. Discussion

The results obtained with the generated deep neural network models indicate the possibility of using this type of machine learning in solving such scientific problems. The network determining the metric age of boys had the lowest prediction errors. MSE error was 31.13, RMSE 5.58, and MAE 2.34. The MAE error means that, in this case, the metric age estimate for boys has an error of 2.34 months. The network assessing boys’ age also had the highest R^2^ coefficient. A detailed summary of the parameters is shown in Table 7.

It should be noted that the first stage of the study produced RBF (Radial Basis Function) networks and did not use all of the developed indicators. Both the first study and the current analysis show that the neural model generated from the learning set determining the tooth and bone parameters of men has a higher accuracy. There is greater inaccuracy in the model determining the metric age of women (Table 8).

All prepared, original indicators were used to generate the models. None of the indicators had less than 0.5 significance. It should be noted that variable X02, X04, and X15 had a large variation compared to other indicators (Table 9). In the future, it is recommended to omit these variables from the network learning process. A summary and characterization of the indicators can be found in Table 10.

The models presented in the study are characterized by high accuracy. Compared with the work of Kim and co-authors [46], the quality of the model determining the age of men and women was 9 percentage points higher. The R^2^ coefficient of the produced model was 0.93; Kim’s model had a quality level of accuracy of 0.84. On the other hand, the difference between the accuracy of the model produced by Farhadian et al. [47] is much higher. The MAE error presented in this team’s study was 4.12 years, while the RMSE error was 4.4 years. The error of the models produced in this work varies depending on the learning set within: MAE from 2.34 to 4.61 months, and RMSE error from 5.58 to 7.45 months. However, it is important to note the difference in the age range of the study subjects, which may have translated into network quality. In Farhadian’s study, the range was between 14 and 60 years of age, whereas in the research presented here, the range was between 4 and 15 years. In turn, Banjšak et al. [48] used convolutional networks to estimate the age of found skulls. This team’s model works with an accuracy of 73%. It should be noted that this team could not know the precise metrical age. Very high accuracy of the produced models was presented in their works by Milošević et al. [49] and Kahaki et al. [50]. However, despite the high values of the indicators defining the networks, the error was measured in years rather than individual months.

Compared to the work of our team [51], it can be seen that the quality of deep neural models is comparable, with an indication for deep learning methods. Table 11 shows the network quality and RMPSE error for each learning set.

The neural model developed in this study is applicable to assess the metric age of only children and adolescents in the age range of 4–15 years. Pantomographic radiographs of patients without systemic diseases and with normal development of the dental buds were used for the study. All images of persons with root canal treatment or extensive fillings in their teeth were also excluded. This is a strong advantage from the point of view of network creation and function. However, from the point of view of diagnostics, the collection should take into account a whole range of cases including anomalies. In addition, the number of teaching cases should increase. The strengths of the paper are the fairly large scope of the dataset and the well-defined cases. The plus side of the research conducted is the use of proprietary indicators that allowed for the development of a new method and the use of neuronal modeling methods. Additionally, note that the artificial neural network simulator used is publicly available under an open license. On the other hand, the disadvantage of works comparing the effect of different technologies is the divergence of quality indicators—different simulators have different measures, and special attention should be given to this.

## 5. Conclusions

The conducted research indicates that neural modeling methods are an appropriate tool for determining the metric age based on the developed proprietary tooth and bone indices. The indicated issue of metric age assessment belongs to the area of medical, biological, and natural sciences and is a highly nonlinear problem. The MAE error of the produced models, depending on the learning set used, is between 2.34 and 4.61 months, while the RMSE error is between 5.58 and 7.49 months. The correlation coefficient R^2^ ranges from 0.92 to 0.96. The produced deep neural models have higher quality already in the first iteration of learning the network using all the developed metrics. It is recommended to prepare deep neural networks based on the set of indicators used in the first stage of the research.

## Figures and Tables

**Table 1:** Parameters of the generated model—age assessment for men and women.

Output-Training Metrics		Output-Validation Metrics		Prediction	
frame size	0.750	frame size	0.250	frame size	Set Female and Male
MSE	14.204018	MSE	153.537238	MSE	49.318690
RMSE	3.768822	RMSE	12.391014	RMSE	7.022727
Nobs	463	Nobs	156	Nobs	619
R^2^	0.979917	R^2^	0.805455	R^2^	0.932248
MAE	2.790147	MAE	10.022930	MAE	4.612949

**Table 2:** Parameters of the generated model—age assessment for men and women.

Variable	Importance	Percentage
X12	1.0	0.0563
X13	0.9163	0.0516
X14	0.8957	0.0504
Sex	0.8892	0.0500
X09	0.8797	0.0495
X16	0.8708	0.0490
X18	0.8456	0.0476
X21	0.8123	0.0457
X05	0.8122	0.0457
X08	0.8104	0.0456
X06	0.8073	0.0454
X10	0.7951	0.0447
X01	0.7924	0.0446
X17	0.7731	0.0435
X03	0.7708	0.0434
X07	0.7656	0.0431
X11	0.7647	0.0430
X15	0.7466	0.0420
X04	0.7280	0.0410
X20	0.7257	0.0408
X19	0.6891	0.0388
X02	0.6786	0.0382

**Table 3:** Parameters of the generated model—age assessment for women.

Output-Training Metrics		Output-Validation Metrics		Prediction	
frame size	0.750	frame size	0.250	frame size	Set Female
MSE	3.232030	MSE	230.694201	MSE	55.486853
RMSE	1.797785	RMSE	15.188621	RMSE	7.448950
Nobs	228	Nobs	68	Nobs	296
R^2^	0.995460	R^2^	0.698284	R^2^	0.923370
MAE	1.387220	MAE	12.132416	MAE	3.855711

**Table 4:** Parameters of the generated model—age assessment for women.

Variable	Importance	Percentage
X07	1.0	0.0559
X18	0.9659	0.0540
X10	0.9658	0.0540
X16	0.9335	0.0522
X11	0.9311	0.0521
X13	0.9306	0.0520
X14	0.9129	0.0511
X05	0.8902	0.0498
X03	0.8760	0.0490
X12	0.8579	0.0480
X17	0.8376	0.0468
X21	0.8376	0.0468
X09	0.8366	0.0468
X06	0.8237	0.0461
X15	0.8022	0.0449
X01	0.8018	0.0448
X08	0.8013	0.0448
X20	0.7462	0.0417
X04	0.7433	0.0416
X19	0.7291	0.0408
X02	0.6581	0.0368

**Table 5:** Parameters of the generated model—age assessment for men.

Output-Training Metrics		Output-Validation Metrics		Prediction	
frame size	0.750	frame size	0.250	frame size	Set Male
MSE	0.287638	MSE	144.669667	MSE	31.130858
RMSE	0.536319	RMSE	12.027870	RMSE	5.579503
Nobs	254	Nobs	69	Nobs	323
R^2^	0.999585	R^2^	0.833466	R^2^	0.957433
Mae	0.360654	Mae	9.627116	Mae	2.340177

**Table 6:** Parameters of the generated model—age assessment for men.

Variable	Importance	Percentage
X08	1.0	0.0616
X18	0.9811	0.0605
X14	0.9335	0.0575
X12	0.9270	0.0571
X09	0.8617	0.0531
X07	0.8517	0.0525
X11	0.8311	0.0512
X13	0.8117	0.0500
X05	0.8063	0.0497
X21	0.7949	0.0490
X10	0.7859	0.0484
X16	0.7778	0.0480
X06	0.7744	0.0477
X01	0.7011	0.0432
X15	0.6937	0.0428
X17	0.6868	0.0423
X04	0.6243	0.0385
X02	0.6234	0.0384
X19	0.6197	0.0382
X03	0.5852	0.0361
X20	0.5502	0.0339

**Table 7:** Parameters of the generated models—prediction of age assessment.

Prediction					
Women and Men Learning Set		Women Learning Set		Men Learning Set	
MSE	49.318690	MSE	55.486853	MSE	31.130858
RMSE	7.022727	RMSE	7.448950	RMSE	5.579503
RMPSE	6.36%	RMPSE	6.86%	RMPSE	4.83%
Nobs	619	Nobs	296	Nobs	323
R^2^	0.932248	R^2^	0.923370	R^2^	0.957433
MAE	4.612949	MAE	3.855711	Mae	2.340177
MAPE	4.10%	MAPE	3.48%	MAPE	2.04%

**Table 8:** Comparison of sensitivity analysis of variables from the first phase of the study and the current study.

	First Investigation			Deep Learning		
Type of Learning Set	Women and Men	Women	Men	Women and Men	Women	Men
Variable	Rank					
*X01*	17	10	18	12	16	14
*X02*	2		11	21	21	18
*X03*	9	9	14	14	9	20
*X04*	1		10	18	19	17
*X05*	21	13	15	8	8	9
*X06*	16	12	17	10	14	13
*X07*	18	1	5	15	1	6
*X08*	11	3	3	9	17	1
*X09*	19			4	13	5
*X10*	14	7	1	11	3	11
*X11*	5		9	16	5	7
*X12*	6	4	4		10	4
*X13*	22	8	7	1	6	8
*X14*	8	2	2	2	7	3
*X15*	3		8	17	15	15
*X16*	10		16	5	4	12
*X17*	13		12	13	11	16
*X18*	4	11	6	6	2	2
*X19*	12	5	13	20	20	19
*X20*	7	6		19	18	21
*X21*	20			7	12	10
*SEX*	15	-	-	3	-	-

**Table 9:** Parameters of the generated models—prediction of age assessment.

Indicator	Type	Min	Max	Mean	Sigma
Sex	Int	0.0	1.0	0.4782	0.4999
Months	Int	52.0	214.0	118.0549	27.0020
X01	Real	0.0230	5.8003	1.1747	0.6631
X02	Real	0.2353	464.5325	10.4758	30.0014
X03	Real	0.0225	4.4507	1.1795	0.5758
X04	Real	0.5192	323.6753	8.2728	20.4937
X05	Real	0.1269	3.9027	1.0722	0.4077
X06	Real	0.0045	1.6925	0.3198	0.2692
X07	Real	1.1691	2.1069	1.3735	0.1631
X08	Real	0.6556	2.6715	1.5773	0.2764
X09	Real	1.1888	2.4927	1.3968	0.1019
X10	Real	1.2049	3.0659	1.9506	0.3763
X11	Real	0.1827	9.3791	4.8461	1.1687
X12	Real	0.1477	6.9919	3.5269	1.1264
X13	Real	0.5363	2.9021	2.2437	0.2184
X14	Real	0.1807	2.7977	1.9363	0.3729
X15	Real	0.2144	43.1420	6.0704	4.6076
X16	Real	0.2337	8.8893	3.1104	0.7433
X17	Real	0.3277	9.7627	4.1757	1.0240
X18	Real	0.0	7.4619	2.9821	0.7819
X19	Real	0.0624	3.4559	0.8438	0.5139
X20	Real	0.0680	4.0761	0.9981	0.6391
X21	Real	0.3125	3.6140	1.1874	0.3937

The Shadow: The most diverse variables.

**Table 10:** Summary of the significance of variables for each learning set and generated model.

Name of the Learning Set	Women and Men	Women	Men
Variable	Importance	Importance	Importance
Sex	0.8892	-	-
X01	0.7924	0.8018	0.7011
X02	0.6786	0.6581	0.6234
X03	0.7708	0.8760	0.5852
X04	0.7280	0.7433	0.6243
X05	0.8122	0.8902	0.8063
X06	0.8073	0.8237	0.7744
X07	0.7656	1.0000	0.8517
X08	0.8104	0.8013	1.0000
X09	0.8797	0.8366	0.8617
X10	0.7951	0.9658	0.7859
X11	0.7647	0.9311	0.8311
X12	1.0000	0.8579	0.9270
X13	0.9163	0.9306	0.8117
X14	0.8957	0.9129	0.9335
X15	0.7466	0.8022	0.6937
X16	0.8708	0.9335	0.7778
X17	0.7731	0.8376	0.6868
X18	0.8456	0.9659	0.9811
X19	0.6891	0.7291	0.6197
X20	0.7257	0.7462	0.5502
X21	0.8123	0.8376	0.7949

**Table 11:** Comparison of the quality of the models from the first phase of the study and the current ones.

Name of the Learning Set	Women and Men		Women		Men	
	First Study	Current Research	First Study	Current Research	First Study	Current Research
**R^2^**	0.9974	0.9322	0.9631	0.9234	0.9993	0.9574
**RMPSE**	3.65%	6.36%	3.36%	6.86%	3.98	4.84%

## Data Availability

The study was not publicly funded. Data are not open-ended.

## Abbreviations

A43	apex of the root of the tooth 43
A45	apex of the root of the tooth 45
A46	apex of the distal root of the tooth 46
A47	apex of the distal root of the tooth 47
C13	top of the crown of the tooth 13
C15	top of the cheek nodule of the tooth 15
C16	top of the distal cheek nodule of the tooth 16
C17	top of the distal cheek nodule of the tooth 17
C43	top of the crown of the tooth 43
C45	top of the cheek nodule of the tooth 45
C46	top of the distal cheek nodule of the tooth 46
C47	top of the distal cheek nodule of the tooth 47
CeD43	distal cervical point of the tooth 43
CeD45	distal cervical point of the tooth 45
CeD46	distal cervical point of the tooth 46
CeD47	distal cervical point of the tooth 47
CeM43	mesial cervical point of the tooth 43
CeM45	mesial cervical point of the tooth 45
CeM46	mesial cervical point of the tooth 46
CeM47	mesial cervical point of the tooth 47
CM16	top of the mesial cheek nodule of the tooth 16
CM17	top of the mesial cheek nodule of the tooth 17
CM46	top of the mesial cheek nodule of the tooth 46
CM47	top of the mesial cheek nodule of the tooth 47
M43	a point on the lower edge of the mandible in the projection of a straight line through points C43 and A43
M45	a point on the lower edge of the mandible in the projection of a straight line through points C45 and A45
M46	point on the lower edge of the mandible in the projection of a straight line through points C46 and A46
M47	a point on the lower edge of the mandible in the projection of a straight line through points C47 and A47
P43	upper point of the pulp chamber of the tooth 43
P45	upper point of the pulp chamber of the tooth 45
P46	top of distal corner of the pulp chamber of the tooth 46
P47	top of distal corner of the pulp chamber of the tooth 47
PCeD43	distal point of the pulp chamber of the tooth 43 in the cervical area
PCeD45	distal point of the pulp chamber of the tooth 45 in the cervical area
PCeD46	distal point of the pulp chamber of the tooth 46 in the cervical area
PCeD47	distal point of the pulp chamber of the tooth 47 in the cervical area
PCeM43	mesial point of the pulp chamber of the tooth 43 in the cervical area
PCeM45	mesial point of the pulp chamber of the tooth 45 in the cervical area
PCeM46	mesial point of the pulp chamber of the tooth 46 in the cervical area
PCeM47	mesial point of the pulp chamber of the tooth 47 in the cervical area
X01	ratio between section |C13C43| and section |C15C45|
X02	ratio between section |C13C43| and section |C16C46|
X03	ratio between section |C13C43| and section |C17C47|
X04	ratio between section |C15C45| and section |C16C46|
X05	ratio between section |C15C45| and section |C17C47|
X06	ratio between section |C16C46| and section |C17C47|
X07	ratio between section |C43A43| and section |P43A43|
X08	ratio between section |C45A45| and section |P45A45|
X09	ratio between section |C46A46| and section |P46A46|
X10	ratio between section |C47A47| and section |P47A47|
X11	ratio between section |CeM43CeD43| and section |PCeM43PCeD43|
X12	ratio between section |CeM45CeD45| and section |PCeM45PCeD45|
X13	ratio between section |CeM46CeD46| and section |PCeM46PCeD46|
X14	ratio between section |CeM47CeD47| and section |PCeM47PCeD47|
X15	ratio between section |C43M43| and section |A43M43|
X16	ratio between section |C45M45| and section |A45M45|
X17	ratio between section |C46M46| and section |A46M46|
X18	ratio between section |C47M47| and section |A47M47|
X19	ratio between section |A43M43| and section |A45M45|
X20	ratio between section |A43M43| and section |A46M46|
X21	ratio between section |A45M45| and section |A46M46|
